# Developing A Baseline Metabolomic Signature Associated with COVID-19 Severity: Insights from Prospective Trials Encompassing 13 U.S. Centers

**DOI:** 10.3390/metabo13111107

**Published:** 2023-10-24

**Authors:** Kaifeng Yang, Zhiyu Kang, Weihua Guan, Sahar Lotfi-Emran, Zachary J. Mayer, Candace R. Guerrero, Brian T. Steffen, Michael A. Puskarich, Christopher J. Tignanelli, Elizabeth Lusczek, Sandra E. Safo

**Affiliations:** 1 Division of Biostatistics, School of Public Health, University of Minnesota, Minneapolis, MN 55455, USAssafo@umn.edu (S.E.S.); 2 Department of Medicine, University of Minnesota, Minneapolis, MN 55455, USA; 3 Center for Metabolomics and Proteomics, University of Minnesota, Minneapolis, MN 55455, USA; 4 Department of Surgery, University of Minnesota, Minneapolis, MN 55455, USAlusc0006@umn.edu (E.L.); 5 Department of Emergency Medicine, University of Minnesota, Minneapolis, MN 55455, USA; 6 Department of Emergency Medicine, Hennepin County Medical Center, Minneapolis, MN 55455, USA; 7 Institute for Health Informatics, University of Minnesota, Minneapolis, MN 55455, USA

**Keywords:** metabolomics, machine learning, COVID-19, targeted metabolic profiling, biomarker identification

## Abstract

Metabolic disease is a significant risk factor for severe COVID-19 infection, but the contributing pathways are not yet fully elucidated. Using data from two randomized controlled trials across 13 U.S. academic centers, our goal was to characterize metabolic features that predict severe COVID-19 and define a novel baseline metabolomic signature. Individuals (n = 133) were dichotomized as having mild or moderate/severe COVID-19 disease based on the WHO ordinal scale. Blood samples were analyzed using the Biocrates platform, providing 630 targeted metabolites for analysis. Resampling techniques and machine learning models were used to determine metabolomic features associated with severe disease. Ingenuity Pathway Analysis (IPA) was used for functional enrichment analysis. To aid in clinical decision making, we created baseline metabolomics signatures of low-correlated molecules. Multivariable logistic regression models were fit to associate these signatures with severe disease on training data. A three-metabolite signature, lysophosphatidylcholine a C17:0, dihydroceramide (d18:0/24:1), and triacylglyceride (20:4_36:4), resulted in the best discrimination performance with an average test AUROC of 0.978 and F1 score of 0.942. Pathways related to amino acids were significantly enriched from the IPA analyses, and the mitogen-activated protein kinase kinase 5 (MAP2K5) was differentially activated between groups. In conclusion, metabolites related to lipid metabolism efficiently discriminated between mild vs. moderate/severe disease. SDMA and GABA demonstrated the potential to discriminate between these two groups as well. The mitogen-activated protein kinase kinase 5 (MAP2K5) regulator is differentially activated between groups, suggesting further investigation as a potential therapeutic pathway.

## 1. Introduction

Despite the success of vaccines, the ability to identify individuals at risk of severe COVID-19 disease is a persistent need. Certain individuals remain at high risk, and healthcare professionals can make better decisions with accurate information about whether or not their patients are at high risk of developing severe outcomes [1]. Metabolomic syndrome has been linked to severe outcomes in COVID-19 [2,3,4], and the underlying metabolic changes induced by this massive proinflammatory infection may have ongoing relevance to other acute viral and bacterial respiratory diseases. Previous metabolomics analyses have attempted to identify at-risk individuals by comparing hospitalized patients with COVID-19 to COVID-negative controls who may or may not be hospitalized for other conditions [5,6,7,8]. The few studies that matched our own report relative concentrations [9,10], or metabolite ratios [11].

To better understand the differences in metabolomic signatures of patients as a function of disease severity, our group conducted a secondary analysis of prospectively collected plasma from patients enrolled in two multicenter randomized controlled trials that evaluated the efficacy of losartan as a treatment in patients with COVID-19 [12,13]. The trials enrolled patients hospitalized with COVID-19 as well as symptomatic outpatients that did not require emergency department or inpatient care, respectively. The objective of this study was to identify potential biomarkers associated with severe COVID-19 in a population of patients with symptomatic disease.

## 2. Materials and Methods

### 2.1. Population

This study was approved by a central institutional review board (outpatient trial: Advarra Pro00042760; inpatient trial: Advarra Pro00042757), and all participants provided written informed consent. Prior to analysis, all data were anonymized to ensure confidentiality.

Patients who participated provided blood samples for one of two multicenter, placebo-controlled randomized clinical trials to evaluate the efficacy of losartan in hospitalized and nonhospitalized patients with COVID-19 [12,13].

Pharmacodynamics and clinical studies did not identify a significant difference in renin–angiotensin–aldosterone system (RAAS) signaling between the losartan and placebo groups, and neither trial demonstrated significant differences in their primary outcomes [12,13]. We also examined PCA score plots in our data, and no patterns in treatment were identified. Given these observations, for the purposes of the present analysis, we merged data from both treatment groups.

The primary outcome was defined based on a standardized accepted scale of severity at 15 days. Specifically, severity was recorded using a modification of the World Health Organization (WHO) ordinal scale. This scale is as follows: (0) death, (1) hospitalized, on invasive mechanical ventilation or ECMO, (2) hospitalized on noninvasive mechanical ventilation or high flow devices, (3) hospitalized requiring oxygen, (4) hospitalized, not on oxygen, and (5) not hospitalized. To handle missing day-15 outcomes, an a priori decision was made to give all outpatients missing a day-15 outcome an outcome of 5 (not hospitalized), and inpatients missing a day-15 outcome their last observed outcome carried forward if they were in the study beyond day 6.

### 2.2. Primary Outcome

A binary outcome, “mild” vs. “moderate/severe”, was constructed to reflect the worst severity experienced by each individual in the studies. This accounts for any outpatients who may have been hospitalized by the end of the study. Individuals that were never hospitalized over the 15-day period were categorized as having “mild” disease, also defined as a WHO score of no lower than 5. “Moderate/severe disease” was defined as being hospitalized within 15 days, or a score of 4 or lower at any point. Our primary outcome coincided with the trial in which each person participated: those categorized as having mild disease were from the outpatient trial, while those categorized as having moderate/severe disease were from the inpatient trial.

### 2.3. Metabolomics Data and Preprocessing

Biospecimens were collected from 133 consenting patients at baseline (day 1 samples, collected at study randomization) and day 15. Of these, 107 individuals had either a known or imputed day-15 COVID outcome. The final dataset with these 107 individuals’ baseline targeted metabolomic measurement and day-15 COVID outcome was used for subsequent analysis. Samples were collected in ethylenediaminetetraacetic acid (EDTA) tubes and plasma was extracted as per trial protocol within 6 h of collection. Plasma was frozen at −80 Celsius and batch analyzed at the end of the trial.

Plasma was analyzed with the MxP^®^ Quant 500 kit by Biocrates (Life Sciences AG, Innsbruck, Austria). A 96-well-based sample preparation device was used to quantitatively analyze the metabolite profile in the samples. This device consists of inserts that have been impregnated with internal standards, and a predefined sample amount was added to the inserts. Next, a phenyl isothiocyanate (PITC) solution was added to derivatize some of the analytes (e.g., amino acids), and after the derivatization was completed, the target analytes were extracted with an organic solvent followed by a dilution step. The obtained extracts were then analyzed by direct injection MS/MS and LC-MS/MS methods using multiple reaction monitoring (MRM) to detect the analytes. Concentrations were calculated using appropriate mass spectrometry software, and data were imported into Biocrates MetIDQ™ software (Oxygen-DB110-3023) for further analysis. The system allows for the measurement of 630 metabolites from 26 compound classes, and the metabolomics data were log2-transformed and scaled to have a mean of zero and a variance of one for each metabolite prior to further analysis.

### 2.4. Statistical Analysis

To compare baseline demographic characteristics in Table 1, chi-square tests [14] for categorical variables and ANOVA [15] for continuous variables were used. We searched for candidate metabolites that differentiate our primary outcome using resampling, Mann–Whitney U-tests [16], partial least squares-discriminant analysis (PLS-DA) [17,18], logistic regression, and Ingenuity Pathway Analysis (IPA) [19]. We compared individuals with mild vs. moderate/severe COVID-19 disease using metabolite expression at baseline. The data processing and analysis steps are summarized in a flowchart shown in Supplementary Figure S1. All the statistical analyses were performed using the R software version 4.1.0 except for the IPA, which was performed with the QIAGEN IPA software (content version 94302991 released on 27 May 2023) [19].

#### 2.4.1. Individual Metabolites Identification

For univariate analysis, Mann–Whitney U-tests were used on each metabolite to compare the levels in patients with mild vs. moderate/severe COVID-19 to identify individual metabolites that differed between the two groups. We used a Benjamini–Hochberg adjustment to control for false discovery rate (FDR) due to multiple hypothesis testing. Metabolites with an FDR-adjusted *p*-value smaller than or equal to 0.05 were considered statistically significant. The univariate analysis was performed as an exploratory analysis to aid data understanding. All variables were kept for subsequent analyses.

Multivariate analyses were conducted in order to identify potential metabolites that discriminated between the two groups. Resampling techniques were used for statistical rigor and robustness. The data were split into 100 training and testing sets with a 70:30 ratio. Stratification by the primary outcome of disease severity was implemented so that the proportions of patients by severity were similar in the training and testing sets. Given the large number of metabolites in the dataset, we implemented supervised filtering for each training split by selecting metabolites whose p-values from Mann–Whitney U-tests were smaller than 0.05 after FDR correction for further analysis. Then, a PLS-DA model with two components was fitted on each of the training data. The variable importance projection (VIP) was used to assess the importance of each metabolite in discriminating between the two groups and to perform variable selection (i.e., identify important biomarkers in discriminating the group of mild and moderate/severe COVID-19). For the PLS-DA model, we averaged the VIP scores from each fit and selected all metabolites with VIP > 1 and appearing in at least 80 out of the 100 splits for further analyses. The PLS-DA model was implemented using the mixOmics [20] package in R.

#### 2.4.2. Generation of a Metabolite Signature

To construct our metabolomic signature, we focused on finding a small set of low-correlated signatures at baseline with the potential to predict the primary outcome using results from the PLS-DA model discussed above. Since the PLS-DA model could select metabolites with relatively high correlation, we followed the approach of Brzyski et al. [21] to select low-correlated metabolites. Metabolites that were moderately to highly correlated (absolute value of the correlation at least 0.3) with this metabolite formed a cluster; the identified metabolite was selected as the representative of this cluster. We next identified the metabolite with the largest VIP score that was not included in the first cluster to form the next cluster of metabolites that were highly correlated with the identified metabolite. We repeated the process of finding metabolites with large VIP score and forming metabolite clusters until all metabolites were in at least one of the clusters. Since our sample size was 107, for sufficient statistical power to detect differences in patients with mild vs. moderate/severe COVID-19, we aimed to develop a parsimonious model that could distinguish these patients with only a few metabolites. Given the potential effect of Lorsatan on metabolites, we fitted a model using the same sets of variables and treatment as a sensitivity analysis to show the robustness of our results.

#### 2.4.3. Pathway Analysis

We used the Ingenuity Pathway Analysis (IPA) software (QIAGEN) for functional enrichment analysis to determine key signaling pathways and upregulators enriched in our list of candidate metabolites. In IPA, we included the fold differences and p-values for each molecule from the differential analyses in order to identify biological functions in our candidate list that are expected to be increased or decreased based on molecular networks curated in the Ingenuity Knowledge Base. For each biological function that is expected to increase or decrease given the observed metabolite expression changes in our dataset, IPA calculates an activation score to infer the likely activation states (“increased” or “decreased”) of biological functions [19]. In particular, given the observed differential regulation of a metabolite (“up” for fold difference > 0, or “down” for fold difference < 0), the activation state of a biologic function is inferred from the edges (relationships) of molecules in the molecular network in the Ingenuity Knowledge Base. An activation score (or activation z-score) greater than 2 (“increased” prediction) or smaller than 2 (“decreased” prediction) is considered significant. Metabolites were matched with the Human Metabolome Database (HMDB) identifiers prior to inputting into IPA. For metabolites without a clear HMDB match, we used LIPID MAPS^®^ IDs provided by Biocrates and matched them with HMDB IDs using the CTS Batch Conversion website from UC Davis (https://cts.fiehnlab.ucdavis.edu/batch, accessed on 19 October 2023). Metabolites that were unavailable from HMDB were excluded from the analysis. The 115 metabolites that were consistently selected by the PLS-DA (at least 80 out of the 100 splits) and VIP > 1 were selected as candidates for the pathway analysis. Nineteen of them were removed because they were unavailable on HMDB when matching.

## 3. Results

### 3.1. Study Population

Table 1 provides an overview of the demographic characteristics of patients included in this study. The group of patients with moderate/severe COVID-19 were more likely to be male (69.23% (n = 27) vs. 54.41% (n = 37)) and were generally older (median: 58.00 (IQR: 48.00–66.00) vs. median: 38.00 (IQR: 26.25–51.00)). When comparing the race of patients with mild with moderate/severe outcomes, we observed that the proportions of individuals identified as Black or African American (43.59% (n = 17) vs. 4.41% (n = 3)) or Hispanic (17.95% (n = 7) vs. 10.29% (n = 7)) were higher in the moderate/severe group, while the percentage identified as white (28.21% (n = 11) vs. 76.47% (n = 52)) was lower. The median BMI was higher in the moderate/severe group (median: 31.96 (IQR: 26.47–35.10)) compared to the mild COVID-19 group (median: 26.69 (IQR: 24.24–31.76)). The proportion of patients randomized to losartan versus placebo was not significantly different.

### 3.2. Identification of Metabolites with Largest Conditional Effect on Moderate/Severe COVID-19 Infection

#### 3.2.1. Univariate Analysis

**Figure 1:**
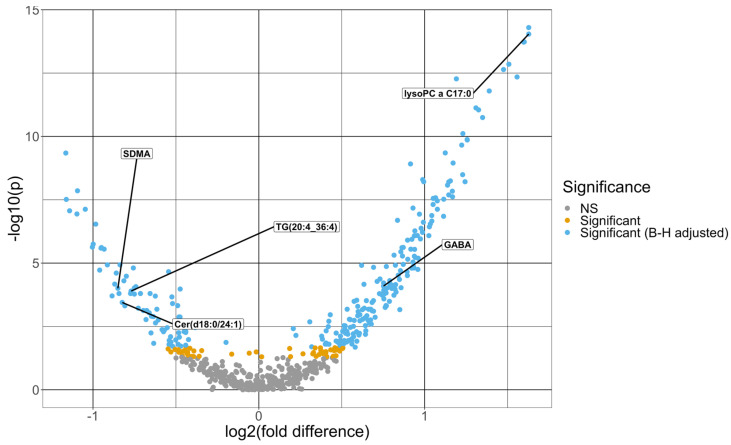
Volcano plot of -log10 (*p*-value) from Mann–Whitney U-tests comparing each metabolite between moderate/severe and mild COVID-19 cases vs. the fold difference in log2 metabolite expression between mild and moderate/severe COVID-19 cases. The fold difference is defined as the difference in the mean log2-transformed and scaled metabolite expression levels between the two groups. The dots in light blue are significant after the Benjamini–Hochberg adjustment, and the ones in the gold are significant before the *p*-value adjustment but not significant after the Benjamini–Hochberg adjustment. The dots in gray are not significant prior to the Benjamini–Hochberg adjustment. The five metabolites that were selected from the multivariate model are labeled in this figure.

The Mann–Whitney U-tests identified 266 baseline metabolites that differed significantly at an FDR level of 0.05, comparing patients with mild vs. moderate/severe COVID-19. Figure 1 shows a volcano plot for the negative log10 *p*-value against fold difference for the baseline metabolites. The fold change difference, defined as the difference between the mean log2-transformed and scaled metabolite expression in the mild group and that in the moderate/severe group [22], ranges from −1.16 to 1.63, where the metabolite with the highest positive and negative fold change difference are lysophosphatidylcholine a C17:0 and beta-aminobutyric acid, respectively. Lysophosphatidylcholine a C16:1 is the metabolite with the lowest *p*-value of 5.04 × 10^−15^. Limiting to metabolites that differed significantly after FDR adjustment, 49 demonstrated a fold change difference magnitude greater than 1, and most (77.6%) of these proteins (38 out of 49) demonstrated higher mean expression in moderate/severe cases. The mean and standard deviation of the baseline metabolites are summarized by severity in Supplementary Table S1.

#### 3.2.2. Multivariable Analysis

**Figure 2:**
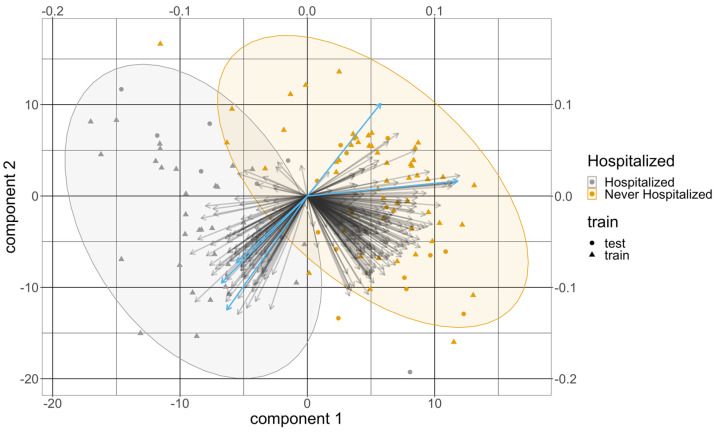
The biplot from the PLS-DA results from one split. The *x*-axis and the *y*-axis at the bottom left are for the score of the two components, and the dots represent the score of these patients colored by their COVID severity. Their shapes represent whether they are from the training set or the testing set. The *x*-axis and the *y*-axis at the top right are for the loadings of the variables. The arrows represent the loadings of the variables, where the five metabolomic signatures were colored in light bule. The order of these five signatures counterclockwise from the x-axis is lysophosphatidylcholine a C17:0, gamma-aminobutyric acid, symmetric dimethylarginine, triacylglyceride(20:4_36:4), and dihydroceramide(d18:0/24:1).

Over the 100 training and testing splits, 479 targeted metabolites at baseline were selected by the PLS-DA model in at least one split. A total of 115 of them were selected at least 80 times with an average VIP greater than 1 from all the splits and were kept for further analyses. Table 2 summarizes the 10 metabolites with the highest average VIP scores from the PLS-DA model. All of them were selected in all of the splits. All the variables chosen for the PLS-DA were significant in the univariate analysis. The average test error rate was 8.1% and the average test AUC was 0.948. Figure 2 provides a representative example of a biplot that overlays the scores and loadings from one split of the PLS-DA results.

#### 3.2.3. Development of a Baseline Metabolomic Signature

**Figure 3:**
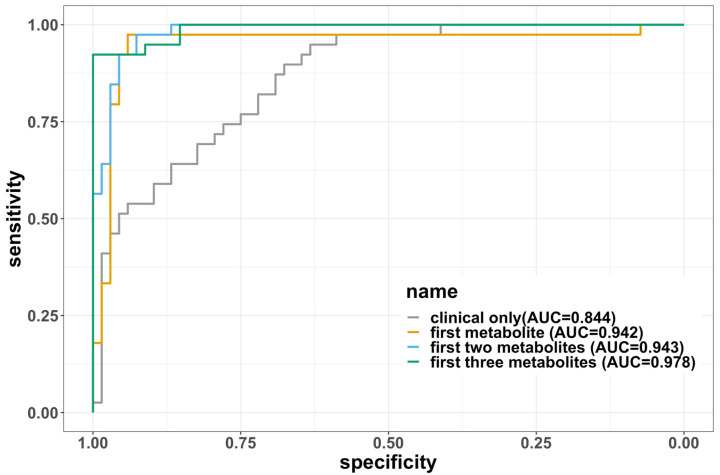
The ROC curves for each of the four logistic regression models fitted. The curves were created using the full dataset without splitting into training and testing, so the areas under the curves (AUCs) are not the same as the average AUCs in the tables. The AUCs are labeled on the legend. The three metabolites are in the order of (1) lysophosphatidylcholine a C17:0, (2) dihydroceramide (d18:0/24:1), and (3) triacylglyceride (20:4_36:4).

Five molecules with low correlation (<0.3) were selected by using the procedure described above. These molecules were (1) lysophosphatidylcholine a C17:0, (2) dihydroceramide(d18:0/24:1), (3) triacylglyceride(20:4_36:4), (4) symmetric dimethylarginine, and (5) gamma-aminobutyric acid. The order was based on the magnitude of their VIP scores from high to low, and we will refer to this order in the following description of the models. The correlations between pairs of molecules were low (absolute value less than 0.3), as illustrated in Supplementary Figure S2. We developed a parsimonious model with these five molecules by adding metabolites with the highest VIP in the list sequentially. The error rates, AUC, sensitivity, specificity, and F1 scores of these models are summarized in Table 3. For reference, we also included the clinical-covariates-only model as a comparison. Due to the limited sample size, the model often diverged for almost all the training splits if more than three molecules were included, so the final model was limited to three metabolites. The final model we selected included lysophosphatidylcholine a C17:0, dihydroceramide (d18:0/24:1), and triacylglyceride (20:4_36:4), which demonstrated excellent performance with an average test error rate of 7.36% and an AUC of 0.978. The comparison among the four models shown in Table 3 can be visualized using ROC curves in Figure 3. Sensitivity analysis that adjusted for treatment status showed that the three metabolites again discriminated between mild vs. moderate/severe disease, with an average error rate of 8.45% and AUC of 0.974. The results of the sensitivity analysis in other models are displayed in Supplementary Table S2.

### 3.3. Pathway Analysis

**Figure 4:**
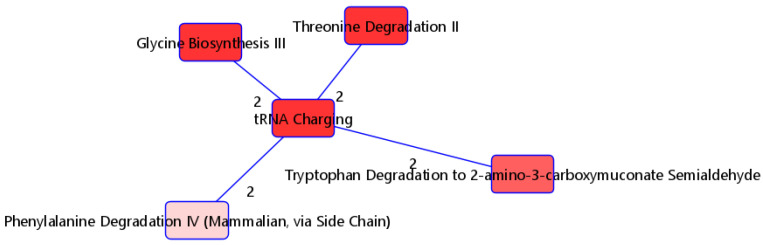
Top five overlapping pathways with at least two shared metabolites. Edge numbers indicate the number of metabolites shared by the two pathways.

The 115 metabolites consistently selected by PLS-DA with high VIP scores (>1) were selected as candidate variables and input into IPA for functional analyses to better understand relevant signaling and metabolic pathways that differ by severity. Nineteen of these metabolites were removed because they could not be matched to any ID in HMDB. We included the fold differences for each molecule from the differential analyses in the IPA. Figure 4 shows the overlapping pathways of the top five canonical pathways with at least two common molecules. The top significantly enriched pathway was the tRNA charging pathway. With the exception of L-phenylalanine, five (L-alanine, L-threonine, L-tryptophan, L-histidine, and glycine) of the six metabolites in our list for this pathway were highly expressed in individuals with moderate/severe disease (Table 4(a)). The distribution of these metabolites for the mild and moderate/severe groups can be visualized on Supplementary Figure S3. Regarding upstream regulators, the kinase MAP2K5, transcription regulator MYC, ORMDL2, and transmembrane receptor CD36 were predicted to be activated. In particular, the kinase MAP2K5 was predicted to be activated, with a z-score of 2.646 and with an overlap *p*-value of 3.92 × 10^−11^. All seven metabolites known to be upregulated by MAP2K5 were observed to be upregulated in our dataset. Furthermore, the transcription regulator MYC was predicted to be activated, with a z-score of 2.333 and an overlap *p*-value of 6.43 × 10^−8^. Eight out of nine molecules known to be upregulated by MYC were also upregulated in our dataset, while the molecule palmitic acid (known to be upregulated by MYC) was downregulated in our dataset (Table 4(b)).

## 4. Discussion

Since the outbreak of the pandemic, there has been an interest in identifying biomarkers associated with the development of severe COVID-19. The primary goal of our study was to develop a baseline metabolic signature associated with the severity of COVID-19 using data from two randomized controlled trials across 13 U.S. academic centers. The multivariate discriminant analysis method identified 115 metabolites that were consistently selected with VIP > 1 to discriminate between mild and moderate/severe disease. A parsimonious model consisting of the three metabolites, lysophosphatidylcholine a C17:0, dihydroceramide (d18:0/24:1), and triacylglyceride (20:4_36:4), highly discriminated between mild vs. moderate/severe COVID-19 with an average test accuracy of 92.6% and AUC of 0.978. Pathways related to amino acids were significantly enriched based on IPA analysis, and the MAP25K regulator was identified as potentially activated, since all the metabolites known to be upregulated by it were upregulated in our data.

PLS-DA modeling demonstrated that LysoPC metabolites strongly differentiate patients with mild COVID-19 from those with moderate/severe disease. Of the top 10 VIP scores, 8 metabolites were LysoPCs. The mechanisms underlying LysoPC’s putative effects on disease development are not yet well understood, and the role it plays in different types of cells varies [23]. In the immune system, the effect of LysoPC molecules includes inducing chemotaxis and thereby regulating immune cells [24]. However, the effect of LysoPC molecules on inflammation remains controversial. Both proinflammatory [23,25] and anti-inflammatory [26,27,28,29,30,31,32] effects of the LysoPC molecules have been reported previously. Our results are in agreement with other studies showing that LysoPC levels are lower in patients with more severe COVID-19 [26,28,29]. A summary of all the LysoPC molecules in our analysis can be found in Supplementary Table S4. In a broader context outside of COVID-19, patients with sepsis have also been shown to have lower levels of LysoPCs than people without sepsis [30]. LysoPCs have been correlated with outcomes in sepsis patients, with higher levels of LysoPCs observed in those treated with appropriate antibiotics and lower levels in those who did not survive [31]. In patients with community-acquired pneumonia, changes in LysoPC levels were also associated with outcomes. LysoPCs were lower on day 1 in nonsurvivors than in survivors and remained unchanged on day 7 in these patients. In survivors, LysoPC levels were increased on day 7 relative to day 1 [32].

Our baseline metabolomic signature identified metabolites that have been associated with COVID-19 severity by other groups and provided external validation and further elucidation of these observations. Our study identified metabolites that include three lipid species (lysophosphatidylcholine a C17:0, dihydroceramide (d18:0/24:1), and triacylglyceride (20:4_36:4)), a modulator of nitric oxide synthesis (symmetric dimethylarginine (SDMA)), and a neurotransmitter (gamma-aminobutyric acid (GABA)). Many metabolomics studies of COVID patients have now reported significant alterations to lipids related to disease severity [10,11,26,28,29,33,34,35], and disruptions to lipid metabolism may persist for years in patients with long COVID [36]. Our data are consistent with these findings. SDMA, meanwhile, is indicative of endothelial dysfunction, and our data are consistent with previous reports that elevated SDMA at the time of hospital admission is associated with poor outcomes [37]. On the other hand, another study only reported elevated asymmetric dimethylarginine, but not elevated SDMA [38]. However, this study represented a comparison of patients hospitalized with COVID vs. hospitalized COVID-negative patients. Our work complements this report by comparing hospitalized patients with nonhospitalized patients, all of whom were COVID-positive, which may explain the difference in findings. Consistent with observations regarding lipid metabolism, patients with long COVID may also see persistent changes in SDMA levels [39]. Finally, decreased GABA levels have previously been observed to be associated with severe disease, while GABA levels were observed to increase over time in those that recovered. In another study, GABA plasma levels allowed for stratification of COVID-19 patients by disease severity [40]. Our data further support these observations.

Functional enrichment analysis of the metabolites consistently selected by our approach revealed a strong enrichment of pathways related with amino acids, including the phenylalanine degradation pathway, glycine biosynthesis III pathway, and threonine degradation II pathway. Many amino acids have previously been demonstrated to be altered in COVID-19 patients relative to healthy controls, particularly phenylalanine [5,6,7,8], though tryptophan and threonine metabolism have also been studied in this context [41,42]. In our work, phenylalanine was elevated in patients with more severe disease, while higher levels of glycine, alanine, histidine, phenylalanine, threonine, and tryptophan were observed in patients with mild disease. IPA analysis suggests that the mitogen-activated protein kinase kinase 5 (MAP2K5), belonging to the MAPK family, is predicted to be activated, with seven out of eight metabolites consistent with MAP2K5 activation. The MAPK family has been implicated in many biological processes, including proliferation, stress response, inflammation, and metabolism, while another recent study demonstrated the levels of MAPK-related biomarkers to be elevated in patients with COVID-19 [43,44]. Our data here indicate that seven metabolites activated by MAP2K5, including cholesteryl pentadecanoate, cholesteryl margarate, cholesteryl linoleate, and cholesteryl oleate, are elevated in patients with moderate/severe disease. Whether this response is pathologic or adaptive cannot be concluded based on our study design, but may represent a potential therapeutic opportunity in COVID-19 or possibly other inflammatory infectious diseases such as pneumonia or sepsis more broadly. Further investigations into these mechanisms, therefore, would seem supported by this study.

Our study provides a simple model that can discriminate between those with mild vs. moderate/severe disease with an accuracy that is at least comparable to previous models [11]. With three metabolite measurements, our model shows an average test AUC of 0.978. The conciseness and efficiency of this model could potentially aid in clinical decisions of whether a patient with COVID-19 will develop a severe outcome. Other strengths include a diverse cohort of patients from multiple sites in the U.S., aiding in generalizability and robustness of our results.

This study acknowledges several limitations. First, the cohort used for the development of the metabolomic signature was derived from two distinct randomized controlled trials. Although the trials were overseen by the same research team with similar inclusion and exclusion criteria, one trial exclusively enrolled symptomatic outpatients afflicted with COVID-19 while the other did not. This divergence might introduce subtle disparities in the underlying data distributions. Our sample did not include COVID-negative patients since the data were from these two trials, so it is unclear whether our results could distinguish COVID-positive and -negative patients. In addition, the trials only admitted patients from a single wave, but the biomarkers for COVID severity may depend on the collection wave [45]. A further study on using patient data collected from other waves can shed light upon to what extent our results can be generalized to different waves of COVID-19. Secondly, our approach relied upon partitioning of our proprietary dataset into training and testing subsets. This raises questions about the degree to which our findings can be extrapolated to a broader population. Thirdly, our sample size was not large enough to perform analysis on subpopulations. Given the heterogeneity of COVID-19, it will be worthwhile to perform such an analysis with a larger sample size to study how the metabolite levels will affect the severity of COVID-19 in different populations in the future. Our sample size also prevented us from including clinical covariates in the final model. Subsequent research may aim to develop a more comprehensive model with both metabolic signatures and clinical covariates (such as age, sex, or BMI) with a larger size data sample. Finally, while the intervention (losartan) did not impact clinical outcomes, it remains possible that there was an effect on biological pathways, though our sensitivity analysis suggests that this did not significantly affect model performance.

## 5. Conclusions

Metabolomic signatures differ significantly by disease severity in COVID-19. A model with three lipid metabolisms most efficiently dichotomized our patient cohort. SDMA and GABA also exhibited discriminatory potential between the two groups. Pathway analysis suggests that the mitogen-activated protein kinase kinase 5 (MAP2K5) is particularly differentially activated between groups and represents a potential therapeutic pathway requiring additional study.

## Figures and Tables

**Table 1:** Demographics of patient samples used in the analysis of the association between baseline metabolome and COVID-19 disease severity. For the continuous variables, the median and the IQR are shown, and for the categorical variables, the count and the proportion are shown.

	Moderate/Severe (n = 39)	Mild (n = 68)	Total (n = 107)	*p*-Value
**Sex**				0.19
Male	27 (69.23%)	37 (54.41%)	64 (59.81%)	
**Age**				
Median [IQR]	58.00 [48.00, 66.00]	38.00 [26.00, 51.00]	47.00 [31.50, 57.00]	<0.001
**Race**				<0.001
Asian	1 (2.56%)	3 (4.41%)	4 (3.74%)	
Black or African American	17 (43.59%)	3 (4.41%)	20 (18.69%)	
White	11 (28.21%)	52 (76.47%)	63 (58.88%)	
Hispanic	7 (17.95%)	7 (10.29%)	14 (13.08%)	
Other/unknown	3 (7.69%)	3 (4.41%)	6 (5.61%)	
**Body mass index (BMI)**				0.004
Median [IQR]	31.96 [26.47, 35.10]	26.69 [24.24, 31.76]	28.40 [24.64, 33.04]	
**Treatment**				0.74
Losartan	21 (53.85%)	33 (48.53%)	54 (50.47%)	
**Diabetes**				0.025
No	29 (74.36%)	63 (92.65%)	92 (85.98%)	
Yes	9 (23.08%)	5 (7.35%)	14 (13.08%)	
Missing	1 (2.56%)	0 (0%)	1 (0.93%)	
**Coronary artery disease**				0.17
No	37 (94.87%)	68 (100%)	105 (98.13%)	
Yes	1 (2.56%)	0 (0%)	1 (0.93%)	
Missing	1 (2.56%)	0 (0%)	1 (0.93%)	
**Hypertension**				<0.001
No	16 (41.03%)	62 (91.18%)	78 (72.90%)	
Yes	22 (56.41%)	6 (8.82%)	28 (26.17%)	
Missing	1 (2.56%)	0 (0%)	1 (0.93%)	
**Atrial fibrillation**				0.046
No	34 (87.18%)	67 (98.53%)	101 (94.39%)	
Yes	4 (10.25%)	1 (1.47%)	5 (4.67%)	
Missing	1 (2.56%)	0 (0%)	1 (0.93%)	
**Pulmonary hypertension**				NA
No	37 (94.87%)	68 (100%)	105 (98.13%)	
Missing	2 (5.13%)	0 (0%)	2 (1.87%)	
**Asthma**				0.74
No	36 (92.31%)	60 (88.23%)	96 (89.71%)	
Yes	3 (7.69%)	8 (11.77%)	11 (10.29%)	
**Chronic bronchitis**				NA
No	38 (97.44%)	68 (100%)	106 (99.07%)	
Missing	1 (2.56%)	0 (0%)	1 (0.93%)	
**Chronic obstructive pulmonary disease (COPD)**				0.010
No	34 (87.18%)	68 (100%)	102 (95.32%)	
Yes	4 (10.25%)	0 (0%)	4 (3.74%)	
Missing	1 (2.56%)	0 (0%)	1 (0.93%)	
**HIV**				NA
No	38 (97.44%)	68 (100%)	106 (99.07%)	
Missing	1 (2.56%)	0 (0%)	1 (0.93%)	
**Uses cigarettes**				0.75
No	37 (94.87%)	62 (91.18%)	99 (92.52%)	
Yes	2 (5.13%)	6 (8.82%)	8 (7.48%)	
**Uses vape products**				NA
No	39 (100%)	68 (100%)	107 (100%)	

**Table 2:** Top 10 baseline metabolites with the highest VIP scores from PLS-DA. Metabolites are ranked according to their average VIP score across the 100 splits. All the metabolites were selected consistently in all the training folds. The fold change difference and the adjusted *p*-value from the univariate analysis are also included in this table for reference.

Targeted Metabolites	Average VIP	Fold Change Difference	Adjusted *p*-Value
Lysophosphatidylcholine a C17.0	1.82	1.63	2.86 × 10^−12^
Lysophosphatidylcholine a C16.1	1.82	1.63	2.86 × 10^−12^
Lysophosphatidylcholine a C18.0	1.79	1.60	2.98 × 10^−12^
Lysophosphatidylcholine a C16.0	1.78	1.60	2.98 × 10^−12^
Lysophosphatidylcholine a C14.0	1.74	1.56	4.03 × 10^−11^
Ceramide (d18.1/24.1)	1.73	−1.14	1.32× 10^−6^
Lysophosphatidylcholine a C18.1	1.68	1.51	1.77 × 10^−11^
Lysophosphatidylcholine a C18.2	1.65	1.48	2.40 × 10^−11^
Lysophosphatidylcholine a C20.3	1.55	1.39	1.12 × 10^−10^
Cholesteryl ester (14:0)	1.50	1.35	9.39 × 10^−10^

**Table 3:** The performance of the logistic regression model with biomarkers only averaged across 100 training and testing splits. The metabolite models do not include clinical covariates. The model with clinical covariate is shown as a comparison.

Variables Included	Error Rate	AUC	Sensitivity	Specificity	F1
Clinical only (age, sex, BMI)	0.265	0.844	0.800	0.628	0.789
First metabolite	0.0668	0.942	0.946	0.911	0.947
First two metabolites	0.09	0.967	0.933	0.87	0.929
First three metabolites	0.0736	0.978	0.938	0.906	0.942

**Table 4:** (**a**) Top five overlapping pathways with at least two shared metabolites. (**b**) Upregulators enriched in IPA. The fold difference is defined as the difference in the mean log2-transformed and scaled metabolite expression levels between the mild and moderate/severe groups. The sign of the fold difference indicates the regulatory direction, where a positive change indicates an upregulation and a negative change indicates a downregulation.

(**a**)																							
**Pathway (*p*-Value)**						**Molecule**						**Fold Difference**						**Adjusted *p*-Value**					
**tRNA charging (2.03 × 10^−5^)**						Glycine						0.917						2.57 × 10^−5^					
						L-alanine						0.961						5.32 × 10^−5^					
						L-histidine						1.244						1.52 × 10^−7^					
						L-phenylalanine						−0.999						1.80 × 10^−5^					
						L-threonine						0.894						1.47 × 10^−4^					
						L-tryptophan						1.114						1.99 × 10^−6^					
**Glycine biosynthesis III (1.16 × 10^−3^)**						L-alanine						0.961						5.32 × 10^−5^					
						Glycine						0.917						2.57 × 10^−5^					
**Threonine degradation II** **(3.94 × 10^−3^)**						L-threonine						0.894						1.47 × 10^−4^					
						Glycine						0.917						2.57 × 10^−5^					
**Tryptophan degradation to 2-amino-3-carboxymuconate semialdehyde (8.23 × 10^−3^)**						L-alanine						0.961						5.32 × 10^−5^					
						L-tryptophan						1.114						1.99 × 10^−6^					
**Phenylalanine degradation IV (mammalian, via side chain)**						L-phenylalanine						−0.999						1.80 × 10^−5^					
						Glycine						0.917						2.57 × 10^−5^					
(**b**)																							
**Predicted upregulators (z-score, *p*-value of overlap)**				**HMDBID**				**Molecule Name**				**Prediction (based on measurement direction)**				**Fold Difference**				**Previous Findings**			
**MAP2K5** **(2.646, 3.92 × 10^−11^)**				HMDB0060057				Cholesteryl pentadecanoate				Activated				1.168				Upregulates			
				HMDB0060059				Cholesteryl margarate				Activated				0.915				Upregulates			
				HMDB0006725				Cholesteryl myristate				Activated				1.349				Upregulates			
				HMDB0010370				Cholesteryl octadecatrienoate				Activated				1.155				Upregulates			
				HMDB0000658				Cholesteryl 9-heaxadecenoate				Activated				1.025				Upregulates			
				HMDB0000610				Cholesteryl linoleate				Activated				0.987				Upregulates			
				HMDB0000918				Cholesteryl oleate				Activated				0.854				Upregulates			
**MYC** **(2.333, 6.43 × 10^−8^)**				HMDB0006725				Cholesteryl myristate				Activated				1.349				Upregulates			
				HMDB0000658				Cholesteryl 9-heaxadecenoate				Activated				1.025							
				HMDB0006736				Cholesteryl eicosatrienoate				Activated				1.034				Upregulates			
				HMDB0000610				Cholesteryl linoleate				Activated				0.987				Upregulates			
				HMDB0010368				Cholesteryl stearate				Activated				0.898				Upregulates			
				HMDB0000929				L-tryptophan				Activated				1.114				Upregulates			
				HMDB0000918				Cholesteryl oleate				Activated				0.854				Upregulates			
				HMDB0000123				glycine				Activated				0.917				Upregulates			
				HMDB0000220				Palmitic acid				Inhibited				−0.952				Upregulates			
**CD36** **(2.219, 2.51 × 10^−3^)**				HMDB0010370				Cholesteryl octadecatrienoate				Activated				1.155				Upregulates			
				HMDB0005435				triaclyglycerol				Activated				0.930				Upregulates			
				HMDB0000658				Cholesteryl 9-heaxadecenoate				Activated				1.025				Upregulates			
				HMDB0000610				Cholesteryl linoleate				Activated				0.987				Upregulates			
				HMDB0000918				Cholesteryl oleate				Activated				0.854				Upregulates			

## Data Availability

The dataset in our study is included in our Supplementary Materials as Supplementary Table S3.

## Supplementary Materials

The following supporting information can be downloaded at: https://www.mdpi.com/article/10.3390/metabo13111107/s1, Figure S1: The flowchart of our project; Figure S2: The correlation plot of the five selected metabolites; Figure S3: The distribution of the metabolites in the t-RNA charging pathway; Table S1: Mean and standard deviation of the significant metabolites in the univariate approach by group; Table S2: Model results from the sensitivity analysis that adjusted for treatment status in addition to the models in Table 3. Table S3: Metabolomics information used in the analysis. Table S4: Mean, standard deviation, and regulating direction of the lysoPC molecules in the analysis.
