# Supplementary figures and images for: Developing A Baseline Metabolomic Signature Associated with COVID-19 Severity: Insights from Prospective Trials Encompassing 13 U.S. Centers
Source: Metabolites. 2023 Oct 24;13(11):1107. doi: 10.3390/metabo13111107 (PMC10672920; doi:10.3390/metabo13111107)

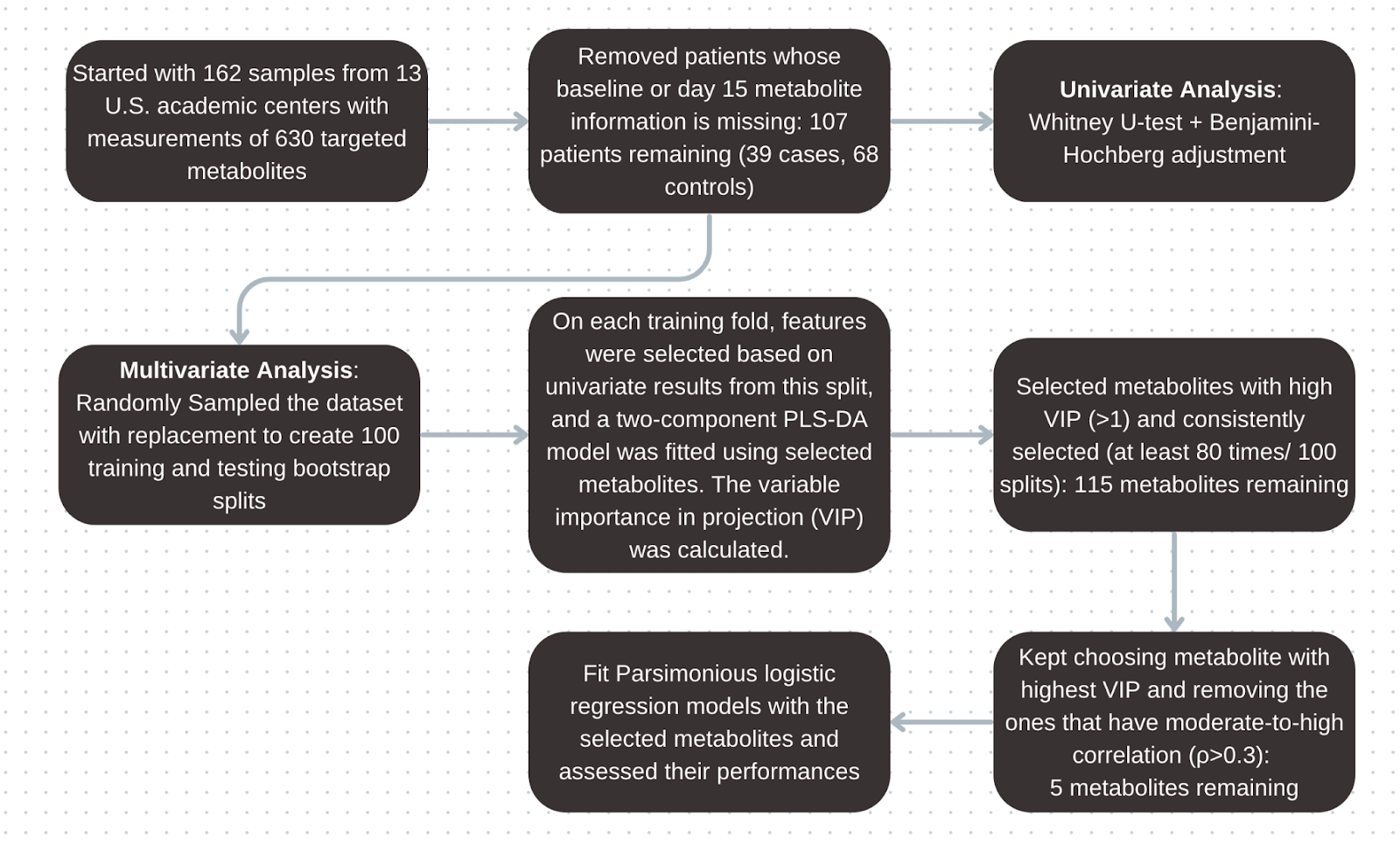

Supplement: Supplementary file 1 [file metabolites-13-01107-s001.zip › Figure S1 flowchart.png]

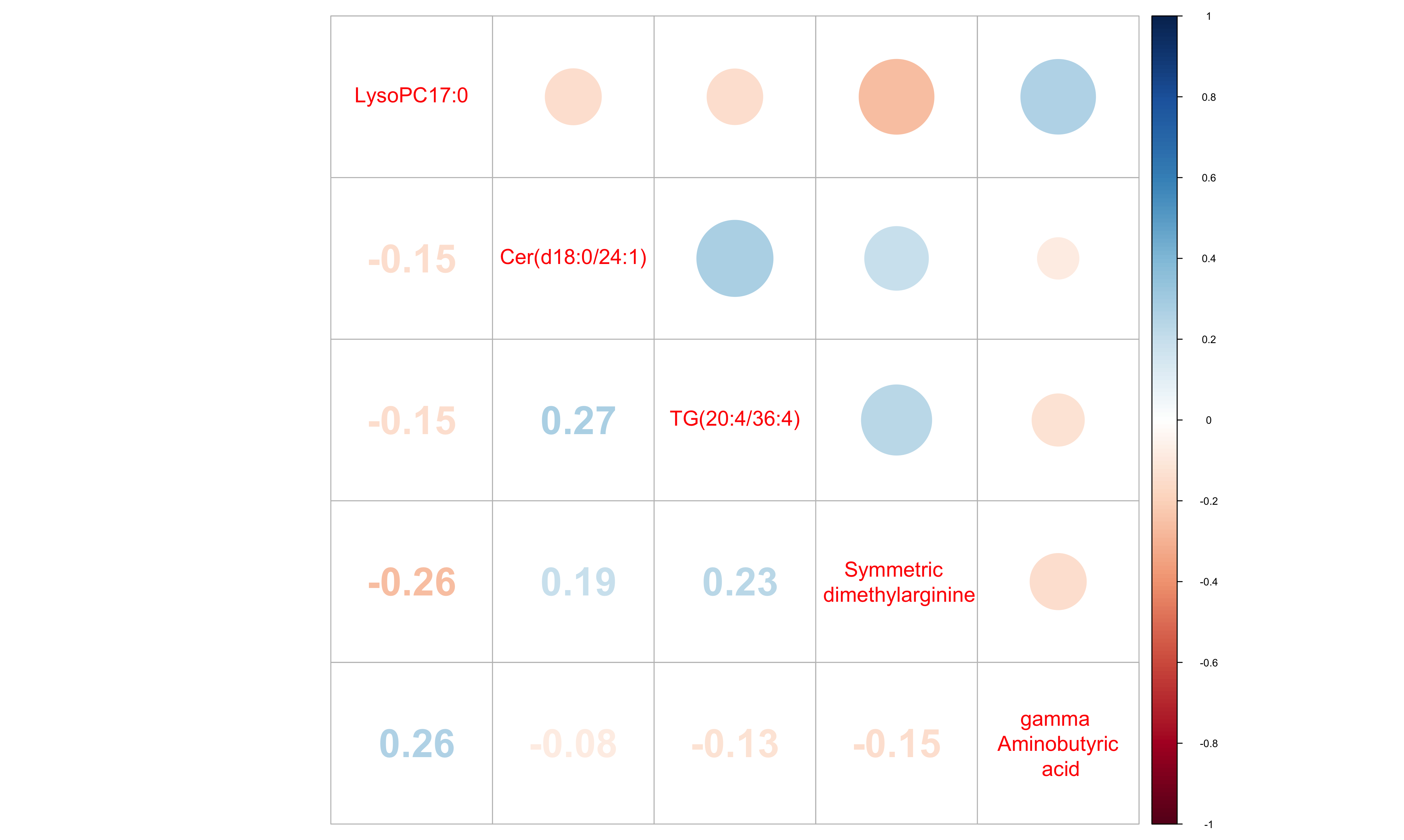

Supplement: Supplementary file 1 [file metabolites-13-01107-s001.zip › Figure S2 correlation plot.png]
